# Targeting the MAPK/ERK and PI3K/AKT Signaling Pathways Affects NRF2, Trx and GSH Antioxidant Systems in Leukemia Cells

**DOI:** 10.3390/antiox9070633

**Published:** 2020-07-17

**Authors:** Ewa Jasek-Gajda, Halina Jurkowska, Małgorzata Jasińska, Grzegorz J. Lis

**Affiliations:** 1 Department of Histology, Faculty of Medicine, Jagiellonian University Medical College, 31034 Kraków, Poland; malgorzata.m.jasinska@uj.edu.pl (M.J.); grzegorz.lis@uj.edu.pl (G.J.L.); 2 Chair of Medical Biochemistry, Faculty of Medicine, Jagiellonian University Medical College, 31034 Kraków, Poland; halina.jurkowska@uj.edu.pl

**Keywords:** ERK, PI3K, reactive oxygen species, NRF2, thioredoxin, glutathione, apoptosis, leukemia

## Abstract

The mitogen-activated protein kinase (MAPK)/extracellular signal kinase (ERK) and phosphatidylinositol 3-kinase (PI3K)/protein kinase B (AKT) signal transduction pathways have been implicated in the pathogenesis of leukemia. The aim of this study was to investigate the effect of the combination of ERK1/2 inhibitor AZD0364 and PI3K inhibitor ZSTK474 on acute lymphoblastic leukemia (ALL) REH, MOLT-4, acute myeloid leukemia (AML) MOLM-14, and chronic myeloid leukemia (CML) K562 cell lines. To evaluate the interactions of the drugs, cells were treated for 48 h with AZD0364 or ZSTK474 alone and in combination at fixed ratios. The combinatorial effects of both inhibitors were synergistic over a wide range of concentrations in REH, MOLT-4, and MOLM-14 cell lines. However, in K562 cells, the effects were found to be antagonistic. Furthermore, AZD0364 and ZSTK474 significantly decreased both ERK1/2 and AKT activation in REH, MOLT-4, and MOLM-14 cells. The results showed that incubation with both AZD0364 and ZSTK474 inhibited cell viability, increased reactive oxygen species (ROS) production, and induced apoptosis in leukemia cells. We observed that combined treatment with AZD0364 and ZSTK474 affected nuclear factor-κB (NF-κB) and antioxidant protein levels: NF-E2-related factor 2 (NRF2), heme oxygenase-1 (HO-1), thioredoxin (Trx), thioredoxin reductase (TrxR), and the reduced glutathione/oxidized glutathione (GSH/GSSG) ratio. These effects were accompanied with decreased antiapoptotic survivin protein level. However, distinct cell line dependent effects were observed. In conclusion, the combination of AZD0364 and ZSTK474 can exert a synergistic anticancer effect in ALL and AML cells, which is associated with the induction of oxidative stress and the involvement of cellular antioxidant defense mechanisms.

## 1. Introduction

The mitogen-activated protein kinase (MAPK)/extracellular signal kinase (ERK), and phosphatidylinositol 3-kinase (PI3K)/protein kinase B (AKT) signaling pathways participate in multiple cellular events, such as cell survival, proliferation, differentiation, motility, and apoptosis [1]. These pathways, however, are frequently deregulated in many cancers, including leukemia, by upstream mutations in receptors, deletions, chromosomal translocations or overexpression of signaling components as well as other different mechanisms [2,3,4]. In order to enhance a therapeutic response in leukemia, new strategies have recently been proposed, including combinations of agents affecting signaling pathways, which inhibit cell proliferation and induce cell death of leukemia cells more effectively. Currently, a wide range of small molecule inhibitors targeting MAPK/ERK and PI3K/AKT pathways have been developed and are being evaluated in preclinical studies. AZD034, a novel selective ERK1/2 inhibitor, has been found to inhibit the growth of human lung cancer cell lines with KRAS mutations [5]. In our previous studies, we have demonstrated that combined treatment of MAPK/ERK pathway inhibitors with topoisomerase II inhibitor is highly synergistic and enhances reactive oxygen species (ROS) production, leading to apoptosis in leukemia cells [6,7]. ZSTK474, a PI3K pan-inhibitor, has been shown to decrease cell survival and induce apoptosis in nelarabine-treated T-lymphoblastic acute leukemia (T-ALL) cells [8]. Moreover, ZSTK474 showed the highest cytotoxic effect in compare with different isoform-selective PI3K inhibitors in T-ALL cell lines [9]. It has been found that ZSTK474 exert synergistic effect when used in combination with ICG-001, a Wnt/β-catenin pathway inhibitor in T-ALL cells [10]. Furthermore, it has been postulated that inhibition of the MAPK/ERK and PI3K/AKT cascades is more effective in inducing apoptosis [11]. The dual inhibition of the MAPK/ERK and PI3K/AKT pathways was found to be synergistic in diffuse intrinsic pontine glioma cells [12]. It has been proposed that simultaneous targeting of MAPK/ERK and PI3K/AKT signaling pathways may be effective in treatment of different types of leukemia without knowledge of the mutation responsible for aberrant activations of these pathways [13]. However, the effect of combined treatment with AZD0364 and ZSTK474 has not been examined in human leukemia cell lines.

The generation of reactive oxygen species after treatment with chemotherapeutic drugs is an important mechanism in drug-induced cytotoxicity of various leukemia treatments [14,15,16]. To avoid the excessive ROS production, cells activate defense antioxidant mechanisms. Antioxidant molecules, however, are deregulated in many cancers, including leukemia, which correlates with increased proliferation, survival, and drug resistance.

The NF-E2-related factor 2 (NRF2), thioredoxin (Trx), and reduced glutathione (GSH) are major cellular antioxidant systems that neutralize the excess of free radicals [17,18,19]. NRF2 is a key transcription factor which controls the expression of many antioxidant proteins [20]. In leukemia, NRF2 has been found to be constitutively activated and involved in cancer cell proliferation [21,22]. Furthermore, it has been reported that the phosphorylation of signaling molecules, such as MAPK and PI3K are strongly associated with the NRF2 activity [21,23]. Heme oxygenase-1 (HO-1), a representative NRF2 target gene, exerts also important redox regulatory functions. HO-1 neutralizes the highly cytotoxic, free radical-producing heme and has been functionally implicated in both acute myeloid leukemia (AML) and chronic myeloid leukemia (CML) [24]. On the one hand, overexpression of HO-1 was detected in samples from AML patients [25]. On the other hand, in HL60 and THP-1 AML cell lines, basal HO-1 expression was shown to be relatively low but easily activated upon oxidative stress [26]. The thioredoxin antioxidant system is composed of several proteins, including thioredoxin, and thioredoxin reductase (TrxR), which play an important role in regulating cellular redox homeostasis, cell growth and apoptosis [27,28]. Thioredoxin-1, a cytoplasmic isoform, has been found to activate the PI3K/AKT pathway, thus regulating many cellular processes, such as cell proliferation, migration, and survival [18,27,29]. Both Trx and TrxR expression have been shown to be elevated in ALL and AML cells, and correlated with cell survival and resistance to apoptosis [30,31]. The GSH system, similarly to previous antioxidant systems mentioned, is capable of removing ROS from leukemia cells. The majority of glutathione is within the cell in a reduced form, but it can be enzymatically converted into its oxidized form GSSG. The ratio between GSH and GSSG is an indicator of oxidative stress [32]. Elevated GSH levels were observed in ALL, AML, and chronic lymphoblastic leukemia (CLL) patient samples [33,34]. As reported previously, increased GSH level was associated with resistance to anticancer drugs [35,36].

In the present study, we aimed to evaluate the synergistic effect of AZD0364, an ERK1/2 inhibitor, combined with ZSTK474, a PI3K inhibitor, on human leukemia cells in vitro. The purpose of this study was to elucidate the possible antioxidant mechanisms underlying this combination treatment.

## 2. Materials and Methods

### 2.1. Inhibitors 

AZD0364 and ZSTK474 were purchased from Selleck Chemicals (Selleckchem, Houston, TX, USA) and Merck Millipore (Billerica, MA, USA), respectively. The stock solutions were prepared at 10 mM in dimethyl sulfoxide (DMSO, Merck Millipore) and were stored at −80 °C until use.

### 2.2. Cell Culture

The human cell lines REH (acute B-cell lymphoblastic leukemia, B-ALL), MOLT-4 (acute T-cell lymphoblastic leukemia, T-ALL), and MOLM-14 (acute myeloid leukemia, AML) were obtained from the German Collection of Microorganisms and Cell Cultures (DSMZ, Braunschweig, Germany). K562 (chronic myeloid leukemia, CML) cells were purchased from the European Collection of Cell Cultures (ECACC, Salisbury, UK). Peripheral blood mononuclear cells (PBMCs) were purchased from Lonza (Basel, Switzerland). All cells were cultured in RPMI-1640 GlutaMax medium containing 10% fetal bovine serum (FBS), 100 U/mL penicillin and 100 µg/mL streptomycin (all reagents from Life Technologies, Carlsbad, CA, USA). Cells were maintained at 37 °C in a humidified 5% CO_2_ atmosphere.

### 2.3. Cell Viability Assay

For cell viability assay, cell lines and PBMCs were seeded in triplicate at 2 × 10^5^ cells/well in a 96-well plate, and then treated with AZD0364 and/or ZSTK474 for 48 h. The CellTiter 96 Aqueous One solution cell proliferation assay (Promega, Mannheim, Germany) was used to assess cell viability according to the manufacturer’s instructions. Briefly, untreated control and AZD0364/ZSTK474 treated cells were washed and resuspended in Hank’s Balanced Salt Solution (HBBS) (100 µl/well) and incubated with CellTiter 96 Aqueous One solution reagent (20 µl/well) for 2 h at 37 °C. Absorbance was measured at 490 nm using Epoch Microplate Spectrophotometer (BioTek Instruments, Winooski, VT, USA). The relative cell viability (%) was expressed as the percentage of untreated control cells.

### 2.4. Drug Interaction Analysis

REH, MOLT-4, MOLM-14, and K562 cells were seeded in triplicate in a 96-well plate (2 × 10^5^ cells/well) and treated with AZD0364 and/or ZSTK474 for 48 h. The combination index (CI) was determined by the method of Chou-Talalay [37] using the CompuSyn software (CompuSyn Inc., Paramus, NJ, USA). Results were expressed as the CI value at the effective dose reducing 50%, 75%, or 90% cell viability (ED50, ED75, and ED90). CI values less than 1 (CI < 1) were considered as synergistic effect, CI equal to 1 (CI =1) as additive and a CI greater than 1 (CI > 1) as antagonistic.

### 2.5. PI3K/MAPK Activity Assay

REH, MOLT-4, and MOLM-14 cells were seeded in triplicate in a 96-well plate (2 × 10^5^ cells/well) and incubated with AZD0364 and/or ZSTK474 for 48 h. The Muse PI3K/MAPK Activation Dual Detection Kit (Merck Millipore) was used to evaluate simultaneously the activation of both phosphorylated AKT and ERK1/2 in cell lines. Briefly, cells were fixed, permeabilized and incubated with a phospho-specific AKT (Ser473)-Alexa Fluor*^®^* 555 and a phospho-specific ERK1/2 (Thr202/Tyr204, Thr185/Tyr187)-phycoerythrin/Cy5 (PECy5) antibodies for 30 min at room temperature in the dark according to the manufacturer’s instructions. The cells were analyzed using a Muse Cell Analyzer and the percentage of cells negative for AKT and ERK1/2 activation, with ERK1/2 activation, with dual pathway activation, and with AKT activation was estimated by Muse analysis software.

### 2.6. Apoptosis Assay

REH, MOLT-4, and MOLM-14 cells were seeded in triplicate in a 96-well plate (2 × 10^5^ cells/well) and treated with AZD0364 and/or ZSTK474 for 48 h. For the assessment of apoptotic cells, the Muse Annexin V and Dead Cell Kit (Merck Millipore) was used according to the manufacturer’s instructions. In brief, cells were re-suspended in RPMI-1640 medium supplemented with 1% FBS and Muse Annexin V and Dead Cell Reagent for 20 min at room temperature in the dark. The cells were quantified using the Muse Cell Analyzer and the percentages of total apoptotic cells were determined.

### 2.7. Oxidative Stress Assay

REH, MOLT-4, and MOLM-14 cells were seeded in triplicate in a 96-well plate (2 × 10^5^ cells/well) and treated with vehicle alone or with AZD0364 and/or ZSTK474 for 48 h. Cell population undergoing oxidative stress was measured using the Muse Oxidative Stress Kit (Merck Millipore) according to manufacturer’s protocol. In brief, cells were re-suspended in Muse Oxidative Stress working solution containing dihydroethidium and incubated for 30 min at 37 °C. The cells were then quantified using a Muse Cell Analyzer and the relative percentage of ROS-positive and ROS-negative cells was estimated by Muse analysis software.

### 2.8. Determination of GSH/GSSG Ratio

Leukemia cells were seeded in triplicate in a 96-well plate (2 × 10^4^ cells/well) and treated with vehicle alone or with AZD0364 and/or ZSTK474 for 48 h. The ratio of GSH/GSSG was determined using the GSH/GSSG-Glo Assay (Promega, Mannheim, Germany) according to manufacturer’s protocol. Briefly, after removal of culture medium, the cells were lysed with either total or oxidized glutathione reagents for 5 min at room temperature. Luciferin Generation Reagent was added to the wells for 30 min at room temperature and after 15 min incubation with Luciferine Detection Reagent, luminescence was measured using Synergy HT Multidetection Microplate Reader (BioTek Instruments) and the ratio of GSH/GSSG was calculated.

### 2.9. Western Blotting

Total protein was extracted from cells using RIPA buffer (Sigma-Aldrich Corp., St. Louis, MO, USA) supplemented with 1X Protease Inhibitor Cocktail (Roche Diagnostic, Basel, Switzerland) followed by centrifugation at 20,000× *g* for 15 min at 4 °C. Protein concentrations were determined by bicinchoninic acid (BCA) assay (Thermo Scientific/Pierce Biotechnology, Rockford, IL, USA). Protein samples (25 µg) were separated by sodium dodecyl sulfate polyacrylamide gel electrophoresis (SDS-PAGE) containing 12% of SDS, and then electrotransferred to polyvinylidene fluoride (PVDF) membranes (Bio-Rad Laboratories, Hercules, CA, USA). The membranes were blocked with 5% non-fat milk and incubated overnight at 4 °C with primary antibodies: anti-NRF2 (1:500, rabbit polyclonal, #16396-1-AP, Proteintech Group Inc., Rosemont, IL, USA); anti-HO-1 (1:800, rabbit polyclonal, #10701-1-AP, Proteintech); anti-NF-κB (1:800, rabbit monoclonal #13586, Cell Signaling Technology (CST), Danvers, MA, USA); anti-Trx (1:1000, rabbit polyclonal, #14999-1-AP, Proteintech); anti-TrxR (1:1000, rabbit polyclonal, #11117-1-AP, Proteintech); anti-survivin (1:1000, rabbit monoclonal #2808, CST); and anti-β-actin (1:1000, rabbit monoclonal, #8457, CST). After washing, the membranes were incubated with goat anti-rabbit secondary antibody conjugated with alkaline phosphatase (1:2000, Proteintech). Bands were developed using 5-bromo-4-chloro-3-indolyl phosphate (BCIP)/nitro blue tetrazolium (NBT, Roche Diagnostic) as substrate. The optical density of the bands was quantified with ChemiDoc MP Imaging System (Bio-Rad Laboratories, Hercules, CA, USA). β-actin was used as the internal control.

### 2.10. Statistical Analysis

GraphPad Prism 5.0 (GraphPad Software Inc. La Jolla, CA, USA) was used for the statistical analysis. The results are represented as the mean ± standard deviation (SD) of three independent experiments. Statistical differences were analyzed by the unpaired two-tailed Student’s t-test with *p* value of < 0.05 as statistically significant.

## 3. Results

### 3.1. Synergistic Interaction between the ERK1/2 Inhibitor AZD034 and PI3K Inhibitor ZSTK474

**Figure 1:**
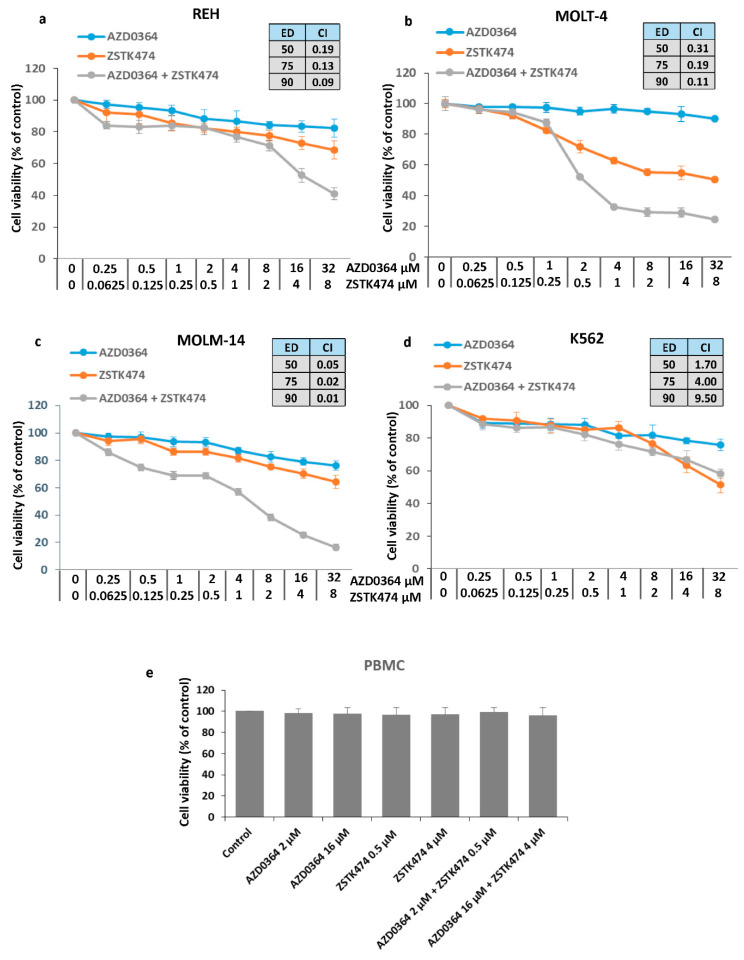
Synergistic anti-proliferative effects of the mitogen-activated protein kinase (MAPK)/extracellular signal kinase (ERK) and phosphatidylinositol 3-kinase (PI3K)/protein kinase B (AKT) pathway inhibitors. (**a**) REH, (**b**) MOLT-4, (**c**) MOLM-14 and (**d**) K562 cells were incubated for 48 h with increasing concentrations of AZD0364 and ZSTK474 alone or in combination with the constant ratio doses. The cell viability was determined by The CellTiter 96 Aqueous One solution cell proliferation assay. The combination index (CI) and effective dose (ED) values were calculated using the Chou-Talalay method described in ″Materials and methods″. (**e**) AZD034 and ZSTK474 alone and in combination did not affect the viability of peripheral blood mononuclear cells (PBMCs). Each value is the mean  ±  SD of three independent experiments.

REH, MOLT-4, MOLM-14, and K562 cell lines were treated with increasing concentrations of AZD0364 (0.25–32 µM) and ZSTK474 (0.0625–8 µM) alone or in combinations for 48 h. In single treatment, AZD0364 demonstrated a little effect on the cell viability in a wide range of concentrations, resulting in survival rate of 75.85 ± 3.65% to 91.12 ± 4.72% in K562 and MOLT-4 cells, respectively. ZSTK474 alone exhibited a dose-dependent growth inhibitory effect and at the highest concentration of 8 µM, the percentage of viable cells ranged from 50.62 ± 4.72% in MOLT-4 cells to 64.34 ± 3.33% in MOLM-14 cells. To determine whether AZD0364 and ZSTK474 interact synergistically, CI analysis was performed. As shown in Figure 1a–c, the effect of combining AZD034 and ZSTK474 at their ED50, ED75 and ED90 was synergistic (CI < 1) in REH, MOLT-4 and MOLM-14 cells. In contrast, in K562 cells, the combinations were found to be antagonistic (CI > 1) irrespective of the effective dose (Figure 1d). Interestingly, the combination of AZD0364 and ZSTK474 did not affect the viability of peripheral blood mononuclear cells (Figure 1e).

Our results show that AZD0364 and ZSTK474 exhibit a synergistic interaction in ALL (REH and MOLT-4) and AML (MOLM-14) cells, but not in CML (K562) cells. Based on the obtained results, REH, MOLT-4 and MOLM-14 cell lines were selected for further experiments and the fixed drug concentrations were used: 16 µM of AZD0364 and 4 µM of ZSTK474 in REH cells, 2 µM of AZD0364, and 0.5 µM of ZSTK474 in MOLT-4 and MOLM-14 cells.

### 3.2. MAPK/ERK and PI3K/AKT Signaling Pathways are Modulated by AZD0364 and ZSTK474

**Figure 2:**
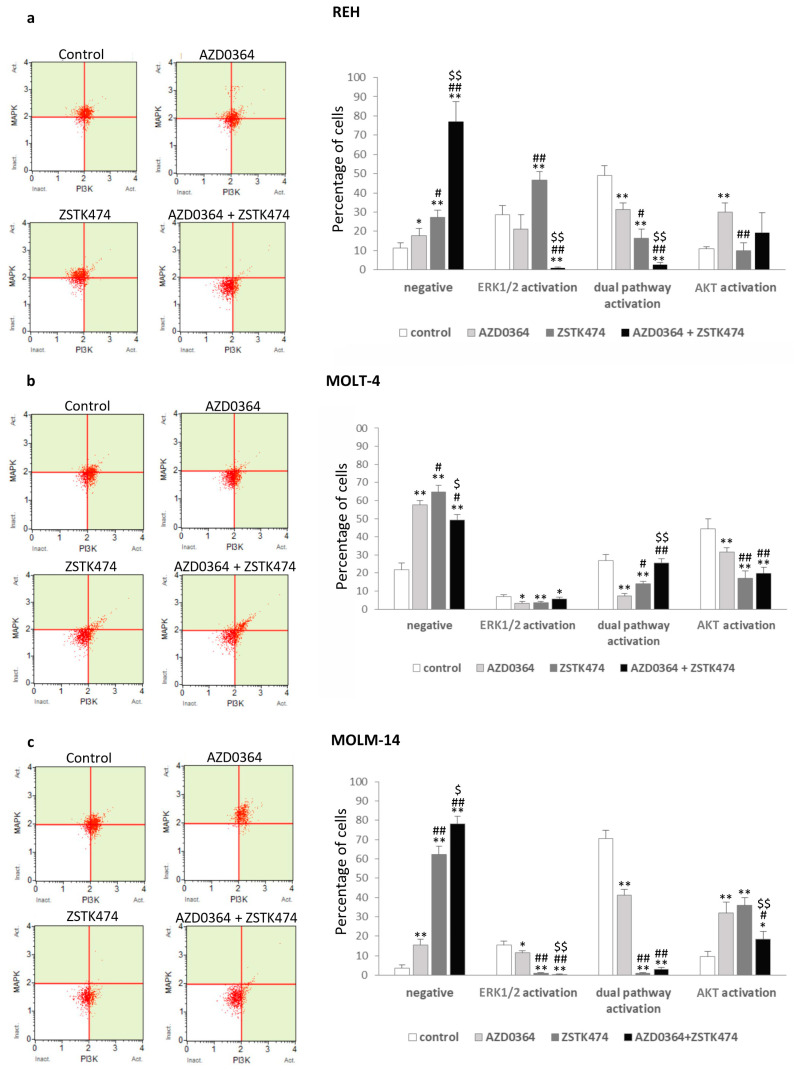
AZD034 and ZSTK474 decreased dual extracellular signal kinase 1/2 (ERK1/2) and protein kinase B (AKT) activation. REH, MOLT-4 and MOLM-14 cells were incubated for 48 h with AZD034 (16 µM for REH, 2 µM for MOLT-4 and MOLM-14 cells) and ZSTK474 (4 µM for REH, 0.5 µM for MOLT-4 and MOLM-14 cells) alone or in combination. ERK1/2 and AKT activation was determined using the Muse PI3K/MAPK Activation Dual Detection Kit. (**a**–**c**) Representative dot plots and graphs of ERK1/2 and AKT activation in REH, MOLT-4 and MOLM-14 cell lines. Each value is the mean  ±  SD of three independent experiments. Significantly different at: * (*p*  <  0.05), ** (*p*  <  0.01) vs. control; # (*p*  <  0.05), ## (*p*  <  0.01) vs. AZD0364; $ (*p*  <  0.05), $$ (*p*  <  0.01) vs. ZSTK474.

To evaluate the status of the MAPK/ERK and PI3K/AKT pathways in leukemia cells treated with AZD0364 and ZSTK474, we analyzed the activation of ERK1/2 and AKT using Muse PI3K/MAPK dual detection kit. As shown in Figure 2a–c, treatment with AZD034 and ZSTK474 alone demonstrated a significant decrease in the percentage of cells with ERK1/2 activation and cells with dual activation of ERK1/2 and AKT, with a simultaneous increase in the number of cells negative for ERK1/2 and AKT activation (from 6.30% ± 0.45% in REH cells to 35.69% ± 3.87% in MOLM-14 for AZD0364 and from 15.76% ± 3.79% in REH cells to 58.52% ± 4.32% in MOLM-14 cells for ZSTK474) as compared with control. The combined treatment with AZD0364 and ZSTK474 showed an increase in the number of cells without ERK1/2 and AKT activation, reaching the highest level in MOLM-14 cells (74.29% ± 3.87%), with a concomitant decrease in the percentage of ERK1/2 and AKT activated cells as compared with control. These results therefore indicate that the combination of AZD034 and ZSTK474 is effective in the inhibition of MAPK/ERK and PI3K/AKT pathways in leukemia cells.

### 3.3. AZD034 in Combination with ZSTK474 Enhances Apoptosis in REH, MOLT-4 and MOLM-14 Cells

**Figure 3:**
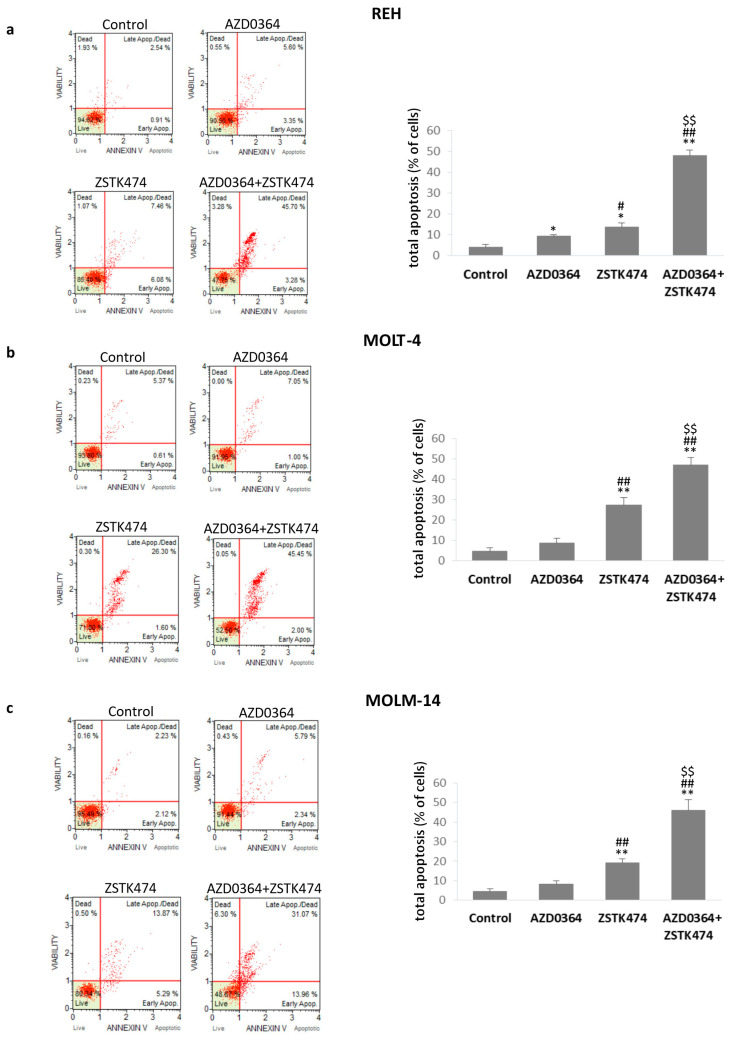
Combination of AZD034 and ZSTK474 enhanced apoptosis in leukemia cells. REH, MOLT-4 and MOLM-14 cells were incubated for 48 h with AZD034 (16 µM for REH, 2 µM for MOLT-4 and MOLM-14 cells) and ZSTK474 (4 µM for REH, 0.5 µM for MOLT-4 and MOLM-14 cells) alone or in combinations. (**a**–**c**) Representative dot plots of Annexin V/7-AAD apoptotic assay and graphs showing the percentage of apoptotic cells. Each value is the mean  ±  SD of three independent experiments. Significantly different at: * (*p*  <  0.05), ** (*p*  <  0.01) vs. control; # (*p*  <  0.05), # #(*p*  <  0.01) vs. AZD0364; $ (*p*  <  0.05), $$ (*p*  <  0.01) vs. ZSTK474.

To determine whether the synergistic effect of the combined treatment could be related to apoptotic cell death, flow cytometric analysis using Annexin V/7-AAD assay was performed. AZD0364 alone had little effect on apoptosis and the percentage of both early and late apoptotic cells did not exceed 10%. However, ZSTK474 alone significantly increased the total apoptotic rate from 13.62% ± 2.12% in REH cells to 27.29% ± 3.87% in MOLT-4 cells. The combined treatment resulted in a higher level of apoptosis compared with either agent alone. There was a modest (less than 2.5-fold) increase in apoptosis in MOLT-4 and MOLM-14 cell lines and a 3.5-fold increase in apoptotic REH cells after the combined treatment compared with cells treated with ZSTK474 alone (Figure 3). These data indicate that AZD0364 and ZSTK474 had little effect on apoptotic cell death when administered alone while they potentiated apoptosis in combination.

### 3.4. AZD0364 and ZSTK474 Increase ROS Production and Alter GSH/GSSG Ratio in Leukemia Cells

**Figure 4:**
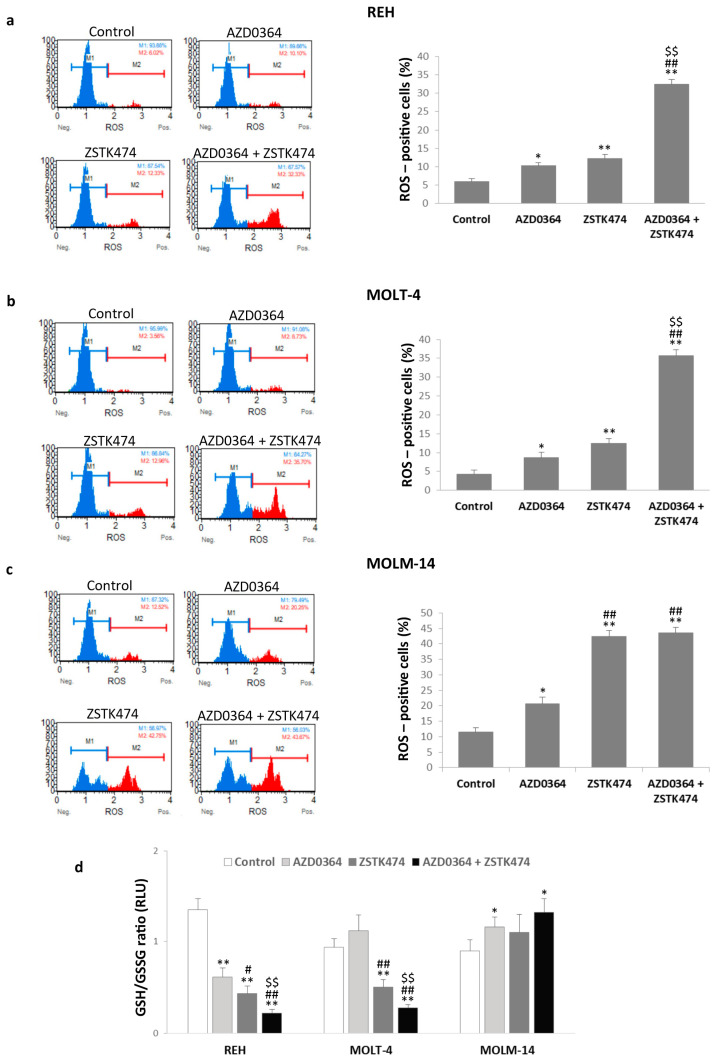
AZD0364 and ZSTK474 enhanced reactive oxygen species (ROS) production and affected reduced glutathione/oxidized glutathione (GSH/GSSG) ratio in leukemia cells. REH, MOLT-4 and MOLM-14 cells were incubated for 48 h with AZD034 (16 µM for REH, 2 µM for MOLT-4 and MOLM-14 cells) and ZSTK474 (4 µM for REH, 0.5 µM for MOLT-4 and MOLM-14 cells) alone or in combination. (**a**–**c**) Representative histograms of ROS-negative (M1) and ROS-positive (M2) cells and graphs showing the percentage of ROS-positive cells. (**d**) The relative GSH/GSSG ratio for untreated control and AZD0364 and ZSTK474 treated REH, MOLT-4 and MOLM-14 cells. Results represent the net luminescence (relative luminescence units, RLU). Each value is the mean  ±  SD of three independent experiments Significantly different at: * (*p*  <  0.05), ** (*p*  <  0.01) *vs* control; # (*p*  <  0.05), ## (*p*  <  0.01) vs. AZD0364; $ (*p*  <  0.05), $$ (*p*  <  0.01) vs. ZSTK474.

Treatment with AZD0364 and ZSTK474 alone resulted in significantly increased production of ROS in all cell lines (Figure 4a–c). The combined treatment with AZD0364 and ZSTK474 markedly potentiated ROS production and the percentage of ROS-positive cells ranged from 32.43% ± 1.38% in REH cells to 43.64 ± 1.74% in MOLM-14 cells. Furthermore, treatment with AZD034 alone resulted in a two-fold decrease of the GSH/GSSG ratio in REH cells, whereas ZSTK474 reduced GSH/GSSG ratio in both REH and MOLT-4 cells (three-fold and two-fold decrease, respectively), compared with the control. The combination treatment resulted in further two-fold reduction in GSH/GSSG ratio in REH and MOLT-4 cells (Figure 4d). In MOLM-14 cells, GSH/GSSG ratio was increased after AZD0364 alone and in co-treatment with both inhibitors (Figure 4d). These findings demonstrate that MAPK/ERK and PI3K/AKT pathway inhibitors promote the generation of intracellular ROS in REH, MOLT-4, and MOLM-14 cells and changes in GSH/GSSG ratio are associated with the leukemia type.

### 3.5. AZD034 and ZSTK474 Affect the Antioxidant and Apoptotic Protein Levels in Leukemia Cells

**Figure 5:**
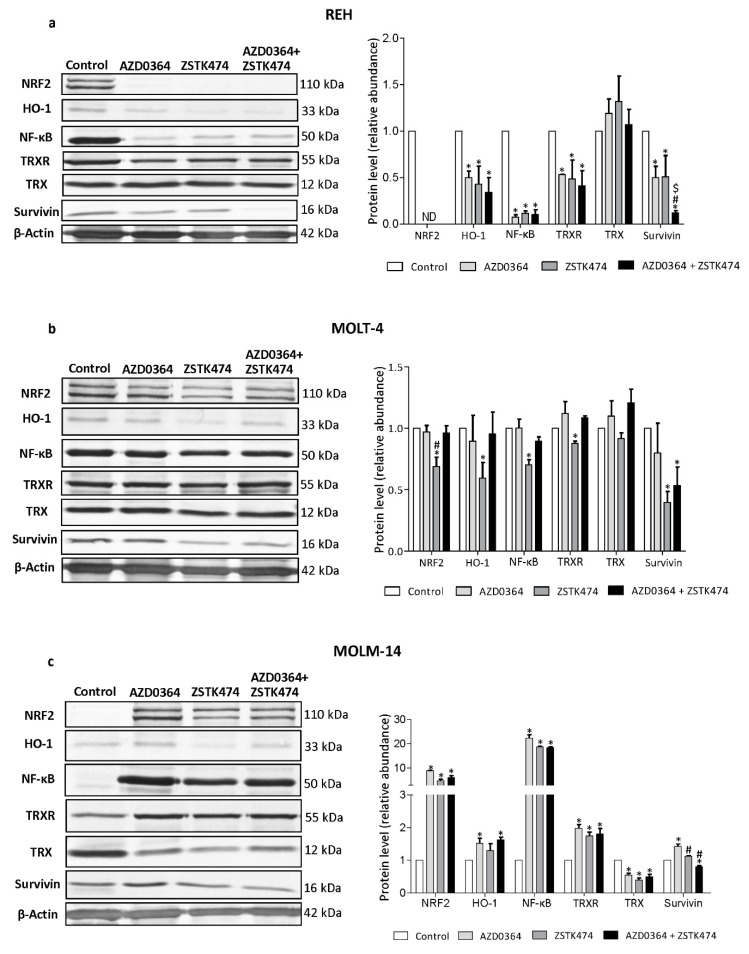
AZD0364 and ZSTK474 affected levels of nuclear factor-κB (NF-κB), NF-E2-related factor 2 (NRF2), heme oxygenase-1 (HO-1), thioredoxin (Trx), thioredoxin reductase (TrxR) and survivin. (**a**) REH, (**b**) MOLT-4 and (**c**) MOLM-14 cells were incubated for 48 h with AZD034 (16 µM for REH, 2 µM for MOLT-4 and MOLM-14 cells) and ZSTK474 (4 µM for REH, 0.5 µM for MOLT-4 and MOLM-14 cells) alone or in combination. The level of indicated proteins was detected by Western blot. β-actin was used as a loading control. Quantification of the proteins was performed by densitometric analysis of the blots and normalized to the internal loading control. Each value is the mean  ±  SD of three independent experiments. Significantly different at: * (*p*  <  0.05) vs. control; # (*p*  <  0.05) vs. AZD0364; $ (*p*  <  0.05) vs. ZSTK474.

To further elucidate the mechanism responsible for AZD0364/ZSTK474 effects, we next investigated proteins involved in antioxidant defense systems and implicated in the regulation of apoptosis by Western blotting. The level of NRF2, one of the key antioxidant and its target protein HO-1 effectively decreased in REH cells, irrespective of the absence or presence of AZD0364 and ZSTK474, whereas in MOLT-4 cells, NRF2 and HO-1 protein levels decreased only after ZSTK0364 treatment. In both REH and MOLT-4 cell lines, AZD0364 and ZSTK474 alone and in combination did not influence the level of Trx, a protein involved in intracellular redox status, whereas TxrR level significantly decreased in REH cells and in ZSTK474-treated MOLT-4 cells. The level of anti-apoptotic survivin was significantly decreased in REH and MOLT-4 cells upon combined treatment with AZD0364 and ZSTK474, compared with untreated control and cells treated with either compound alone. In REH cell line, Western blot analysis also showed a significant decrease in the level of NF-κB protein, a key regulator of pro-survival factors, compared with untreated control and cells treated with AZD0364 and ZSTK474 alone, whereas in MOLT-4 cells, NF-κB protein levels decreased only in ZSTK474 treated cells (Figure 5a,b). In contrast, the MOLM-14 cell line showed the increased level of NRF2, HO-1 and NF-κB proteins after AZD0364 and ZSTK474 treatment alone and in combination. Moreover, the level of TrxR was increased in MOLM-14 cells and the amount of Trx was markedly decreased upon treatment with either inhibitor alone and in combination. Survivin protein level was significantly decreased after combination treatment, compared with control and AZD0364 treated MOLM-14 cells (Figure 5c). These results suggest that, depending on leukemia cell type, the combination of AZD0364 and ZSTK474 shows its pro-apoptotic effect by decreasing or increasing some cytoprotective and regulatory proteins, including NRF2, HO-1, and TrxR. Moreover, these data might provide also an explanation for the involvement of NF-κB and survivin proteins in the synergistic effect of AZD0364 and ZSTK474 combination.

### 3.6. AML MOLM-14 Cell Line Demonstrates the Lowest Level of NF-κB, NRF2, HO-1 and TrxR Proteins, While CML K562 Cells Show the Highest Level of HO-1 and Survivin Compared with ALL and AML Cell Lines 

**Figure 6:**
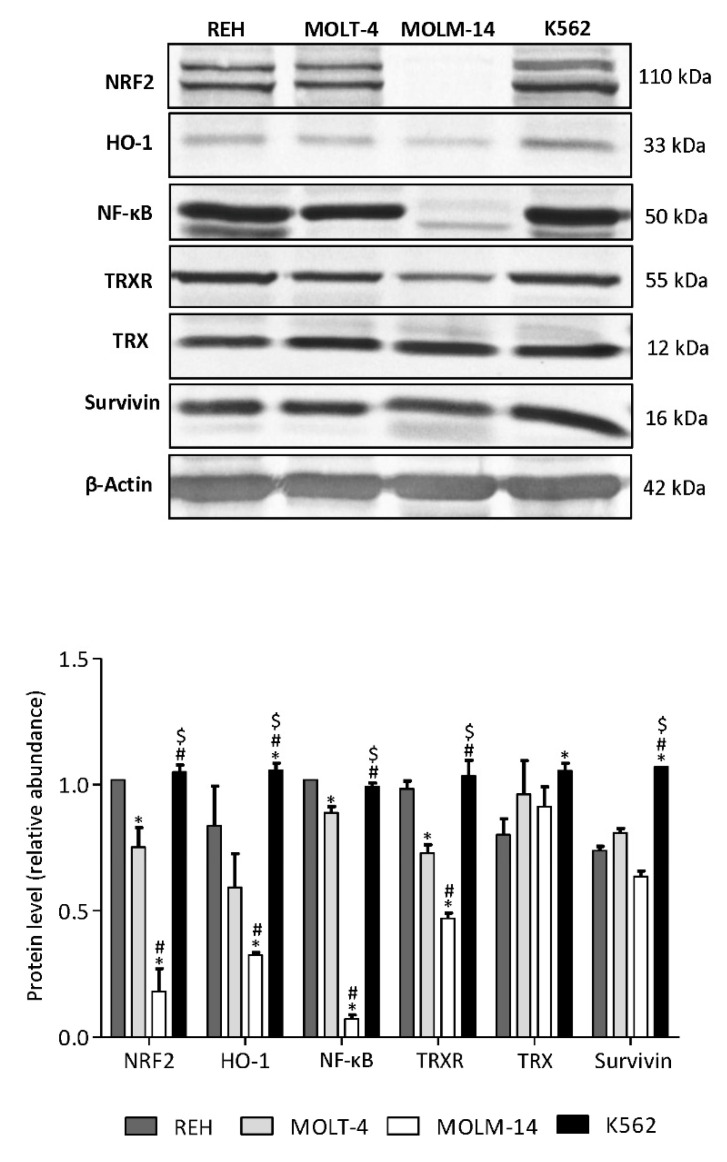
The basal levels of the indicated proteins in untreated control REH, MOLT-4, MOLM-14 and K562 cell lines. The level of the proteins was detected by Western blot. Quantification of the proteins was performed by densitometric analysis of the blots and normalized to the β-actin loading control. Each value is the mean  ±  SD of three independent experiments. Significantly different at: * (*p*  <  0.05) vs. REH; # (*p*  <  0.05) vs. MOLT-4; $ (*p*  <  0.05) vs. MOLM-14.

Furthermore, we determined the protein levels in untreated control leukemia cell lines. MOLM-14 cells showed the lowest basal level of NF-κB, NRF2, HO-1 and TrxR proteins, compared with REH, MOLT-4 and K562 cell lines. CML K562 cells demonstrated significantly higher levels of HO-1 and survivin proteins in compare with ALL (REH and MOLT-4) and AML (MOLM-14) cell lines. Furthermore, the amount of NRF2 and Trx protein levels were significantly higher in K562 cells in compare with MOLT-4 and MOLM-14 cells (Figure 6). These results may suggest that the resistance of K562 cells to combination treatment may be due to the increased levels of antioxidant HO-1 protein and anti-apoptotic survivin.

## 4. Discussion

The pathogenesis of leukemia involves abnormal activation of various signaling pathways, that control cell proliferation, differentiation, metabolism and survival. Thus, targeting these pathways may provide the basis for the development of new therapeutic strategies. In the present study, we have shown that a combination treatment using ERK1/2 inhibitor AZD0364 and PI3K inhibitor ZSTK474 enhanced anticancer effects in leukemia cells by reducing cell viability, and inducing ROS production and apoptosis. Moreover, AZD0364 and ZSTK474 synergistically decreased cell survival in a concentration-dependent manner in all cell lines. In addition, we showed that the CI values of all matched combinations were < 1 in REH, MOLT-4, and MOLM-14 cells, which suggest evident synergistic cytotoxic effects of the combination treatment. However, such an effect was not observed in K562 cells and the CI values were > 1, indicating the antagonism of the drug combination. More interestingly, although both inhibitors showed synergistic inhibitory effect on viability of leukemia cells, they were non-toxic for healthy mononuclear blood cells, what is highly desirable in leukemia therapy. In the present paper, both ERK1/2 and AKT has been found to be at high basal level in REH, MOLT-4 and MOLM-14 cell lines, which is in accordance with the previous data showing that these pathways may be strongly activated in leukemia cells [2,3]. We have shown that AZD0364 and ZSTK474 significantly reduce ERK1/2 and AKT activation in leukemia cells and this effect can be still observed after combined treatment with both inhibitors. Apoptosis is the most well-studied programmed cell death characterized by series of morphological and biochemical changes. One of the most important events in apoptosis is mitochondrial dysfunction and enhanced ROS formation [38]. Thus, high levels of reactive oxygen species may be involved in cancer therapy by inducing apoptosis. Previous study has shown that a standard AML chemotherapy induces an increase in ROS level in leukemia cells [39]. It should be noted that cell response to treatment with combination of drugs can depend on several of factors. In some cases, sensitization of cells to a drug or potentiation of another drug action is induced by ROS formation [40]. Cancer cells can regulate ROS levels by enhancing endogenous antioxidant mechanisms, however they may be more prone to the accumulation of ROS than normal cells. The oxidative stress increased by exogenous ROS generating agents has an effect of selectively killing cancer cells without affecting normal cells [41,42]. In our study, we observed that apoptotic cell death induced by combinatorial treatment with both AZD0364 and ZSTK474 is related to enhanced ROS production in ALL and AML cell lines. 

Altered expression of antioxidant proteins or their deregulation is observed in different types of leukemia, which suggests the importance of these proteins to the disease. NRF2 and its principal target protein HO-1 are frequently increased in several types of tumors, including leukemia, leading to the resistance of cancer cells to chemotherapy [22,43]. Furthermore, it has been reported that, in leukemia cells, NRF2 expression is regulated by other transcription factors, including NF-κB [44]. In our study, AZD0364 and ZSTK474 alone and in combination markedly decreased NF-κB and NRF2 protein levels, which was accompanied by a significant decrease in HO-1 and TrxR protein levels in B-ALL REH cells. However, these effects were observed in T-ALL MOLT-4 cells only following single treatment with ZSTK474. These results suggest that the treatment of ALL REH and MOLT-4 cells with studied inhibitors affects the reduction of NF-κB protein level, which in turn decreases antioxidant protein levels, such as NRF2/HO-1 and TrxR, and leads to the excessive generation of ROS. These findings are in accordance with our previous study, in which the reduction of NF-κB protein level and an increase in ROS production was observed after treatment with ERK2 inhibitor VX-11e and voreloxin in leukemia cell lines [7]. Furthermore, in the present study, we showed that combined treatment with AZD0364 and ZSTK474 reduced GSH/GSSG ratios in both REH and MOLT-4 cells, which may also lead to overproduction of ROS and induction of apoptosis. Previous studies have described that altering glutathione antioxidant system is associated with apoptotic cell death in cancer cells [45]. 

With increasing oxidative stress, other mechanisms are also induced in cells, resulting in a disruption of the redox balance, induction of apoptosis and cell damage. Survivin, an anti-apoptotic signaling protein, regulates cell proliferation and survival [46]. High levels of survivin are detected in many hematological malignancies, including leukemia [47]. Furthermore, it has been found that survivin expression is regulated through MAPK/ERK-dependent mechanisms and induced through PI3K/AKT signaling pathway in leukemia cells [48,49]. In the present study, survivin levels were significantly decreased in REH and MOLT-4 cells after combined treatment with AZD0364 and ZSTK474. Previously, we and others have shown that the synergistic pro-apoptotic effect of MAPK/ERK inhibitors combined with other drugs was accompanied by reduced expression of survivin [7,50]. Moreover, it has been demonstrated that NRF2 regulates survivin expression in endometrial cancer cells [51]. Thus, our results suggest that treatment with AZD0364 and ZSTK474 may trigger an oxidative stress mechanism, leading to decreased cell viability and induction of apoptosis.

In contrast, the present study demonstrated that treatment with both AZD0364 and ZSTK474 enhanced ROS production resulting in the increase of NF-κB and NRF2/HO-1 pathway proteins in AML MOLM-14 cells. Interestingly, we have observed that MOLM-14 cells showed the lowest basal level of NF-κB, NRF2, HO-1 and TrxR proteins in compare with REH, MOLT-4 and K562 cells. MOLM-14 is the FMS-like tyrosine kinase 3-internal tandem duplication (FLT3-ITD) mutated cell line. Constitutively active FLT3-ITD contributes to enhanced proliferation and survival of myeloid progenitor cells and is associated with the poor prognosis and aggressive behavior of AML [52]. It was demonstrated that FLT3-ITD activates the MAPK/ERK and PI3K/AKT pathways which results in the inhibition of apoptosis [53]. Moreover, it was shown that FLT3-ITD mutations cause increased ROS production [52]. Other findings have revealed that treatment with stress inducers resulted in the increased NRF2 activation in AML cell lines [26]. Similarly, a previous study reported the upregulation of NRF2 in MOLM-14 cells treated with dimeric naphtoquinones by enhancing ROS levels [54]. Our results may indicate that upon oxidative stress, antioxidant mechanisms are activated in treated MOLM-14 cells, as evidenced by increased of NRF2/HO-1 pathway as well as GSH/GSSG ratios and TrxR protein levels. However, this response was insufficient to protect MOLM-14 cells from oxidative stress, leading to decreased viability and apoptosis. The amount of Trx was reduced in MOLM-14 cells following AZD0364 and ZSTK474 treatment. It has been shown that upon oxidative stress, cysteine thiol groups in Trx may be oxidatively modified which results in protein inactivation [55,56]. Moreover, it was reported that Trx is expressed in proliferating cells and downregulated in apoptotic ones [57]. Our results have also shown that survivin level decreases in MOLM-14 cells after combined treatment. Other studies reported that FLT3-ITD regulates survivin expression via PI3K/AKT leading to uncontrolled proliferation of myeloid precursor cells. Furthermore, survivin expression was demonstrated to be higher in AML patients samples with FLT3-ITD, compared with samples lacking this mutation [58]. It has been shown that survivin is downregulated in response to increased oxidative stress in the human A549 adenocarcinoma cell line, bladder, and breast cancer cells [59,60,61]. 

Altogether, the above result show that depending on the cell type and regulatory mechanisms, the excessive ROS generation may be associated with decreased antioxidant levels in leukemia cells. On the other hand, the resulting oxidative stress in turn can activate antioxidant proteins as a response.

Furthermore, we wanted to elucidate the possible resistance mechanism of chronic myeloid leukemia K562 cells during combined treatment with AZD0364 and ZSTK474. In the present study, we have demonstrated that K562 cells, show the highest basal level of HO-1 in compare with ALL and AML cells. It has been described that leukemic oncogenes may affect the transcription or activity of antioxidant proteins in leukemic cells. The CML K562 cells carry the breakpoint cluster region–Abelson leukemia virus (Bcr/Abl) oncogene which inhibits apoptotic cell death and is responsible for the resistance to different anticancer drugs [62]. Interestingly, Bcr/Abl was found to enhance HO-1 expression in K562 cells. Moreover, it has been demonstrated that HO-1 is involved in Bcr/Abl-dependent survival of CML cells [43]. In addition, it was reported that Bcr-Abl also enhances the activation of NF-κB and promotes survivin expression in CML cells [63,64,65]. Further studies should be performed in order to understand the molecular mechanisms of action of antioxidants in different types of leukemia.

## 5. Conclusions

Taken together, these results represent an important link between targeting MAPK/ERK and PI3K/AKT signaling pathways and redox control mechanisms in leukemia cells. We have shown that AZD0364 acts in synergy with ZSTK474 in the inhibition of ALL and AML cell viability. Furthermore, it was associated with the excessive ROS production by affecting antioxidant systems and proteins involved in apoptotic cell death. These effects, however, were dependent on the type of leukemia. Within this context, it is reasonable to suggest that the combination of MAPK/ERK and PI3K/AKT inhibitors can affect the antioxidant systems, and therefore might have the potential to enhance anticancer effect in ALL and AML cells.
